# *“Without a mother”*: caregivers and community members’ views about the impacts of maternal mortality on families in KwaZulu-Natal, South Africa

**DOI:** 10.1186/1742-4755-12-S1-S5

**Published:** 2015-05-06

**Authors:** Lucia Knight, Alicia Ely Yamin

**Affiliations:** 1 School of Public Health, University of the Western Cape, Bellville, South Africa; 2 Harvard University, Health Rights of Women and Children Program, François-Xavier Bagnoud Center for Health and Human Rights, Boston, USA

**Keywords:** maternal mortality, orphaned children, family impact, South Africa

## Abstract

**Background:**

Maternal mortality in South Africa is high and a cause for concern especially because the bulk of deaths from maternal causes are preventable. One of the proposed reasons for persistently high maternal mortality is HIV which causes death both indirectly and directly. While there is some evidence for the impact of maternal death on children and families in South Africa, few studies have explored the impacts of maternal mortality on the well-being of the surviving infants, older children and family. This study provides qualitative insight into the consequences of maternal mortality for child and family well-being throughout the life-course.

**Methods:**

This qualitative study was conducted in rural and peri-urban communities in Vulindlela, KwaZulu-Natal. The sample included 22 families directly affected by maternal mortality, 15 community stakeholders and 7 community focus group discussions. These provided unique and diverse perspectives about the causes, experiences and impacts of maternal mortality.

**Results and discussion:**

Children left behind were primarily cared for by female family members, even where a father was alive and involved. The financial burden for care and children’s basic needs were largely met through government grants (direct and indirectly targeted at children) and/or through an obligation for the father or his family to assist. The repercussions of losing a mother were felt more by older children for whom it was harder for caregivers to provide educational supervision and emotional or psychological support. Respondents expressed concerns about adolescent’s educational attainment, general behaviour and particularly girl’s sexual risk.

**Conclusion:**

These results illuminate the high costs to surviving children and their families of failing to reduce maternal mortality in South Africa. Ensuring social protection and community support is important for remaining children and families. Additional qualitative evidence is needed to explore differential effects for children by gender and to guide future research and inform policies and programs aimed at supporting maternal orphans and other vulnerable children throughout their development.

## Background

South Africa has policies of free maternity care and legal abortions, almost universal antenatal care coverage (97%) and most deliveries are facility-based [1,2]. Despite this the maternal mortality ratio (MMR) in 2010 was an estimated 300 deaths per 100,000 live births [3]. While debate exists over the accuracy of this figure it seems that there is consensus that it is high considering the socio-economic and policy context in South Africa [4-6]. While high, recent reductions in the MMR [5,6] suggest improvements in reproductive and obstetric services but these are slow and relatively small. The results suggest that although most maternal mortality is preventable the risk for South African women is still large and necessitates action at multiple levels to reduce rates of mortality and morbidity [1,7,8]. Experts agree that HIV is likely to contribute greatly to this MMR; 2010 estimates suggest that 60% of deaths from maternal causes were attributed to HIV [3] with HIV-infected mothers significantly more likely to die during childbirth than HIV-negative mothers [9]. While HIV may result in an overall increase in adult mortality [4], evidence suggests positive women are at both direct and indirect risk of maternal mortality, [7,10] and that antiretroviral treatment (ART) may also contribute to increased risk during pregnancy [11-13].

The primary motivation to date for improving maternal health is the prevention of maternal mortality as supported by a human rights based approach, which in South Africa is supported by a strong constitution and legislation about rights to accessing health care [14,15]. This paper expands on this to explore the consequential impacts that maternal mortality has for the children and families of women within the South African context. The paper aims to provide an in-depth qualitative exploration of the family level impacts resulting from a failure to stem, what is largely preventable, maternal mortality within this context, including evidence for the way in which families cope with these impacts [10]. The evidence from this study begins to suggest that in addition to the human rights argument for investment in the prevention of maternal mortality that in assessing the socio-economic impacts the need to address maternal health is framed within the broader issues of development [16].

In a very high prevalence context such as South Africa the role of HIV introduces complexities because it becomes difficult to differentiate between the impacts of maternal death, likely from HIV-related causes, at any stage and maternal mortality as defined as a death during pregnancy and childbirth [17,18]. Despite these difficulties we argue that there is still value in this study which provides evidence for the impacts of maternal mortality for the family even if it only proves to confirm existing evidence for the impacts of a maternal death at other times.

An analysis of child survival in a similar site in rural South African highlights the extreme risks that surviving children whose mother’s die of maternal causes face. Infant death after maternal mortality shows the relative significance of a healthy mother for children’s survival. Surviving infants of women who died of maternal causes (WHO defined) were 15 times more likely to die than those whose mothers survived and were also more likely to die than those whose mothers died late maternal deaths or death at other times [19]. The results also highlight the fact that the survival rates of infants born to mothers who die are remarkably high (83%) and this points to the potential long-term impacts of orphaning during infancy for the child and their family [19].

The impacts of HIV-related adult mortality were well researched within the early 2000’s and a developing literature within South Africa about the long-term impacts of orphaning as a result of HIV exists [20-22]. Few studies have explored the impacts of maternal mortality specifically, in a high prevalence context where HIV is a likely cause of death, on the well-being of the surviving infant, older children and the family. This paper aims to provide a detailed, context-specific account of the impacts of maternal mortality on families within rural KwaZulu-Natal. In so doing we hope to highlight the need to refocus on investment in the prevention of maternal mortality, particularly the large burden of preventable causes, but also assess the potential policies and interventions which may work to both support and assist families in coping with the impacts of maternal mortality in the South African context.

## Methods

This qualitative research conducted in South Africa is part of a four-country mixed methods study (including Tanzania, Ethiopia and Malawi) on the impacts of maternal mortality on living children. This study was conducted in Vulindlela, KwaZulu-Natal province outside of the city of Pietermaritzburg. The community includes both rural and peri-urban settlement types and the population is primarily Zulu (the largest ethnic group in South Africa). KwaZulu-Natal is the most populous province in South Africa with 10.3 million people [23]. It also has the highest prevalence of HIV, approximately 40% of women testing at antenatal clinics in the surrounding district were positive in 2011 [24]. The population within this community experiences high levels of poverty and unemployment; but these are comparable to similar rural or peri-urban communities in the rest of the country [25].

The study adopted the WHO definition of maternal mortality as the death of a woman during pregnancy, childbirth, or within 42 days of pregnancy termination, from a pregnancy related cause. The study employed an experienced and resident community liaison officer, to use snowball sampling to locate families affected by maternal mortality. Families were screened to verify that death resulted from maternal causes. Enrolled families included at least one child orphaned after the mother’s death. An attempt was made to include families with orphaned children of varying ages and time since the mother’s death, to reflect a range of impacts.

The study sample included 22 families of women who were identified as dying from maternal causes (one family lost two sisters to maternal mortality). The respondents were mostly grandmothers (40%), and except for a partner, a brother, and a husband, all respondents were women including mothers-in-law, sisters and nieces. The families captured in the study were observed by field staff to be as poor as or poorer than other households within their neighbourhood and many noted that they survived on government social welfare and informal work. Over half of the deaths from maternal causes occurred amongst women who were between the ages of 20-29 years old (57%). Family member interviews addressed the general characteristics of the family for socioeconomic context; circumstances surrounding maternal mortality, the impacts on the children and family; and availability and accessibility of services for maternal orphans.

In-depth semi-structured interviews were conducted with key community stakeholders with likely insights into maternal mortality or working with affected families. Stakeholders included teaching and nursing staff, community councillors, community care workers, church officials, representatives from NGOs and social workers. In addition, 7 focus group discussions (N=60) were conducted to examine community perceptions about maternal mortality, implications for orphans and available services. Data collection occurred between March and August 2013.

An experienced research team conducted the interviews in *isiZulu*; these were then audio-recorded, transcribed and translated. Quality assurance was provided through independent review of a sample of translations to ensure accuracy and standardisation. Each interview and focus group took between 1.5 and two hours to complete. The data was coded utilizing an iterative framework analysis approach, guided by an initial set of codes, based upon the study research questions, which were expanded upon as themes emerged in repeated analyses and readings of the data. Two research staff coded each of the transcripts, discussing and editing themes as they emerged. All analyses were conducted in NVivo 10.

Study protocols were approved by the Harvard School of Public Health Institutional Review Board and the Human Sciences Research Council Research Ethics Committee in South Africa. Informed consent was read verbatim by the research coordinator and all participants indicated consent through a signature. Family member and focus group participants also received ZAR 110 (10.50 USD) for their participation.

## Results

The results for this study are presented at two key levels. The impacts at the family-level, even if these may be felt by an individual such as the caregiver, with social and possibly economic repercussions for the household. The impacts for the children directly, the child-level impacts are presented in terms of the potential impacts over the child’s life-course from infancy into childhood and finally to adolescence, drawing on the life course model to assess the impact at these various life stages.

### Family level impacts of maternal mortality

Caregiving for children left behind

The primary impact of maternal mortality was the orphaning of her children, either the surviving infant or older children, or in many cases both. Orphaned children, whether single (maternal orphans) or double orphans (maternal and paternal orphans), were almost all cared for by female family members. Childcare in this context is traditionally seen as the responsibility of women and participants confirmed that the role was often taken on by grandmothers.

… when mothers who stay with children die, children are left with their grandmothers. Eh, usually people who are good at taking care of the children are their grandmothers. (Social worker)

The general inadequacy of men in providing care and taking primary responsibility for raising children was noted in family and stakeholder interviews, and focus group discussions.

Men find raising a child very difficult. He now has to be in the mother’s shoes and play the role of a mother, and that can be very difficult for a man. (Antenatal clinic sister)

Even where a father was present the expectation was not for him to provide care and he was supported by women who took on this responsibility.

Many family members felt obligated to provide care following a death from maternal causes; and in addition to taking on the roles of caregivers, some family members took on a social parenting role.

[The child] thinks her uncle is her father and the wife of her uncle is her mother. [The aunt] doesn’t have children of her own, [the child] just calls them mommy and daddy. (Grandmother respondent)

Respondents felt that despite facing difficulties the care provided to children was adequate. Nevertheless there was universal recognition that the presence of the mother would have been better for all concerned, especially the children left behind.

There is a huge difference because in our culture it is said that a person that has a mother is better than the one that doesn’t have. (Community focus group)

The responsibility for caring was extended throughout the life course of the child and because of the maternal nature of the death in almost all cases within the study the responsibility started in very early infancy for the index child.

Moreover, the responsibility of caring for children, particularly infants, had potential subsequent impacts for the women who took it on. Some had to take on new employment or informal work in order to care for the child/ren. Others were limited in their ability to look for or take on new work as a result of caring responsibilities. A number even had to give up employment or informal work to care for children, particularly infants who required intensive care.

I am no longer working… I had to stop working because I was told to fetch this child from the hospital [, when the mother died]… and I lost my job. [Now] I work one day [a week]. (Grandmother caregiver)

For older women, grandmothers or great-grandmothers, caregiving responsibilities bore potential repercussions for physical health. As this woman describes, the obligation and desire to provide care to her infant grandchild outweigh the challenges.

I was supposed to carry him on my back and be up and down with the child when he is not well…some people thought of help[ing] me by taking the child to an orphanage. They said I’m too old, that I am not able to raise a child, and I said, I’m still alive, I will try to look after this baby. (Grandmother caregiver)

Despite fulfilling this role as well as possible, caregivers, like the one above, recognized their own inadequacies and were not always prepared for this long-term responsibility, though they were acutely aware of the gap left in the child’s life by their mother’s death.

Complex family arrangements and responding to maternal mortality

South African families are characterised by complexity in the form of fluid membership because of a history of economic and political upheaval [26,27]. This complexity is compounded by low rates of marriage or co-habitation and high rates of extra-marital fertility [28]. This means that besides men being perceived as inadequate caregivers, fathers were sometimes missing from the families in which women died.

Absent fathers were not necessarily missing completely; limited co-habitation or marriage before death meant that men were often not present or residing with their children. Traditional norms dictate that maternal orphans be cared for by the woman’s family, especially if this was where they had been living before her death [29].

They all stay here at home, which is something we agreed on, that after her death we would like them all to be here at home, because they were staying with her here while she was still alive. We decided we will use what we have to give them life… So nothing should be impossible to us. (Deceased’s brother)

Families with multiple orphaned siblings may also have multiple fathers, increasing the complexity in orphaned children’s care and relationships with their fathers. Some fathers were completely uninvolved either because the child’s family did not know who or where he was or because he chose not to be involved. In a number of the cases within the study the mother was very young when she died and had not yet provided information about the father to her relatives, this increased the complication of the situation by limiting the number of people responsible for surviving infants and children.

I don’t know the father of this kid my child. I thought I was still going to get time to sit down with her and ask her. Kids can get pregnant and hide it from you and you only see when they go to deliver the child…To find out who was responsible for this. (Grandmother caregiver)

Regardless of the relationship and complexities in the family, and where they were known, men were obligated to provide financial support for the orphaned child or children. This support, when received, was often very important both for the child and the household in which the child resided.

It means I can say that his father is good…he helps me, although he is far because he lives [away] but he helps me. (Grandmother caregiver)

In lieu of the father, the paternal family may be called on to provide financial or in-kind support for the children, although this cannot always be guaranteed.

On this point of families and the feeding of orphans, by rights the family of the father has to help, but in most cases it becomes the problem of the mother’s family, because this is where the child is born…Usually fathers stay very far away. It’s as if when the person who actually connected the two family’s dies that’s the end of their responsibility. (Older women’s focus group)

In such cases, even the paternal family may provide primary support and care for the children; this was often the case if the parents were married before the death or where the paternal family were in a better position to provide this support. Even when children lived and were cared for by the maternal family, fathers or the paternal family often remained involved, as this maternal grandmother describes.

[the paternal] grandmother, they used to come in December and take the child… every [summer holiday], she would take the child and spend quality time with him without a problem. She sends money on [a] monthly basis. (Grandmother caregiver)

The extended family more generally also provided financial and other support for the child(ren) and assistance to the children’s primary caregiver.

I would say everybody [in the family] contributes because even my brother, if I inform him the granny has not collected her pension and the baby needs this and that he normally come with it on Friday when he comes home for the weekend, his [new] wife also contributes. (Aunt caregiver)

Household coping with death from maternal causes

Care and support of orphaned children is potentially burdensome and was costly for the already vulnerable families who were all surviving with limited income and employment.

…because now [the orphaned children] take from us. I can’t even afford the fruits now because of them.

In addition, the women’s loss was felt in many ways within her household. Not just the fact that she was lost as primary caregiver of her children but also that she was in almost all cases an important financial contributor to the household, supporting herself, her children and others within the household. In addition, women provided support to their families and were responsible for any number of small household chores and activities.

She was a good person. You would have found this place amazingly clean. Dusted. Even if the house is not beautiful but it would be cleaned… she would do the washing and clean. (Grandmother caregiver)

Despite struggling to do so, most of the study families managed to rally both human and financial resources from the extended family network to absorb the extra burden of care. Families were also supported by the community.

…I would say there was really not a big problem in the way the children were provided for with their needs from both [their mother and father’s] families. From the neighbours, I would say I have received some help from them as well. I’ve seen sometimes [the children] have some money and when I ask where they got it from they say it was given by the granny from next door. (Father caregiver)

In addition to support from the family and community, access to social grants was very important in helping families support themselves and the orphaned children. Although targeted at orphaned children, the foster care grant provided by the South African government [30] was not received by many of the children in the study. Reasons included having a living father (an exclusion criteria), as explained below.

We even tried social workers nothing worked. I only managed to register the child in March. They told me there that I wouldn’t get foster care because their father is alive. (Aunt caregiver)

The complicated application process was another reason; particularly the paperwork and proof of death requirements that were barriers for certain families.

You see the granny will try [to apply for the foster care grant] and there will be some hiccups that will make her stop… sometimes they will ask for the letter from the councillor [as proof of father’s absence] and she will not have it at that time… she doesn’t have all the documents with her. (Aunt caregiver)

Social grants targeted generally to children or older people within vulnerable households were particularly important for families and children affected by maternal mortality as illustrated by the quote below, which also highlights the consequences of the loss of the mother and her income for this family.

[The mother] was the one who was taking care of the children. She was working, [when] she was paid I would call her and inform her that we don’t have food and she would give us money and I would go to town and buy the needs… The impact [of her death] has been big, [the children] are suffering; they have to wait for the [child] support grant to get their needs [met] or else they should wait for the granny to get her pension money, sometimes the granny will have debts to pay and she won’t have enough [money]. (Cousin caregiver)

The following quote from a grandmother carer of surviving children highlights the relative importance of grant income for supporting both the household and the children.

In fact my child I did get support because I sometimes ask myself what would I be doing if I did not get grant from the government to support all these children, because from the R1000 I’m get for pension it was not going to be easy, I think God help me to get support so that it can be easy for me to raise these children. (Grandmother caregiver)

Another grandmother carer had similar arguments about the role of the grant.

I survive through my pension money…They got birth certificate from the hospital so that we can be able to apply for the child support grant…That young daughter of mine is the one who collect their grants. She then buys school needs, after getting the money she will make enquiries of what do they need and she will buy that. (Grandmother caregiver)

Funds from grants were particularly important for the care of infants who had special needs that were an additional expense for the family. In some cases families were lucky enough to benefit from the receipt of formula milk support from the clinic.

We bought a large formula milk and nappies with our pension money. I took [the child] to the clinic [for a fever], I told the nursing sister that she has lost her mother, then sister wrote me a letter ensuring I will get formula milk from the clinic for 6 months. So after 6 months when they stop giving me formula, I will be able to buy it using the child support grant. (Grandmother caregiver)

Access to social grants was very important in terms of helping the household cope financially—both with the death of a mother, and to facilitate adequate care for the child or children left behind.

### Impact of death on children through the life-course

Maternal mortality has differential affects for the surviving children depending on the age (immediate effects) and life-stage (longer-term effects) of children at the time of death. For example, orphaned infants failed to receive the long-term benefits of bonding with and being breastfed by their mother [31]. Despite this, and possibly because they are helpless and require comprehensive care, infants’ families report relatively good care, despite financial difficulties and possible opportunity cost to carers.

As children progressed from infancy to early childhood a number became sick. While some were normal childhood illnesses, a few children were diagnosed with HIV after their mother’s death. In many of these cases the mother’s HIV status was not known to her family; consequently neither was the risk to the child until he or she became ill.

When he was sick I took him to [the clinic] and explained everything to them … they created a [clinic] card for me that I will use always when the child is sick. It was difficult because [they found that] the child is HIV-positive. He goes to the hospital, and takes the pills now. (Grandmother caregiver)

Access to treatment for prevention of vertical transmission meant that not all children of HIV-positive mothers were infected. In other cases the diagnosis of the child preceded the mother’s death. Chronic long-term illness has implications for the child and their development, including potential stigma as well as for the family and particularly the primary caregivers who become responsible for young children or infants who require lifetime treatment, adequate nutrition and regular contact with health services. Failure to diagnose illness at birth meant that in many cases the child was already unwell and symptomatic at diagnosis increasing pressure and potentially influencing longer-term morbidity and mortality of the child.

The emotional impacts of a mother’s death seem to be felt more acutely for older children who had known their mother in life.

…it’s very important because they lose someone that they have perhaps stayed with for years and someone that they know very well. So they are very saddened. So they would need to go for counselling, so they can get assistance. (Pastor at local church)

Some of the respondents also noted that sometimes family members were not equipped to support children in dealing with their mother’s death, because they were simultaneously dealing with the loss themselves and in many cases also caring for infants or younger children who require significant inputs in terms of time.

Which means the child will not get the love of the mother. Yes the granny may give him love but it will never be the same as that of the mother, because even the granny herself has been badly affected by this. (Grandmother caregiver)

These emotional difficulties sometimes manifested themselves in difficult behaviour or poor schooling outcomes which caregiving family members sometimes struggled to control or know how to deal with.

…her child had a problem he would just scream loud and cry, even at night, if we ask what is wrong he won’t give you an answer. He was a child who has been doing well at school but since his mother passed he is failing at school. You will help him with his schoolwork and you will be comfortable that he is clear but at school if they ask him he will get it wrong and if we ask what happened he will tell you that he forgot. We do realize that he misses his mother… (Aunt caregiver)

Regardless of age at death, the respondents felt that children as they reach school-going age and interact with peers becoming increasingly aware of their circumstances and that those people caring for them were not their mother. At this key stage children felt their mother’s absence and lack of guidance and support acutely.

They do attend school though their performance is not the same and that sometimes prompts you to follow up on the problem of the child … (Deputy school principal)

Older children and those whose mothers died during adolescence were of the greatest concern to caregivers, as the emotional adjustment to life without a mother was perceived to be greater for them. As noted by this stakeholder:

… for the one who is fourteen and knew her mother, the pain will hit her the most… the young one can be adopted by a family member and never have to miss the mother’s love. (Primary school educator)

It was acknowledged by others that even those who had been brought up by someone else and never really known their mother may experience difficulties once they reach adolescence. Adolescence is observed as a time where rebellion and behavioural problems may become particularly problematic for children orphaned through maternal mortality.

The one who is a teenager may even be rebellious because they do things they were not doing while the mother was still alive and the aunt does not tolerate that… (Mixed community focus group)

Older female children were of particular concern because of the fact that they were perceived to be at increased risk of abuse, early sexual debut, teenage pregnancy and HIV acquisition.

…she has a boyfriend now and her ears are closed, she no longer listens. I am trying but I cannot reach her… she is stubborn you see. I want her to learn and finish [standard] 10 (final year of schooling, grade 12) but now since she has a boyfriend… (Grandmother caregiver)

It is important to note that the risks noted above for female adolescents also increases their risk of maternal morbidity or mortality and therefore result in a cycle of impacts from a failure to address maternal health issues.

## Discussion

These findings highlight that a death from maternal causes has potentially complex and multi-layered impacts on both their surviving children throughout the life course and for the families and individuals tasked with their care, within a rural and peri-urban context in KwaZulu-Natal, South Africa. Despite being relatively poor, families absorbed orphaned children and while women are expected to fulfil traditional gender roles and provide care the contribution of men cannot be discounted. The priority of all caregivers is satisfactory care of the child, and while cultural practices may dictate otherwise, traditions that determine the placement of children were adapted to ensure the best care of the child. While the role of the family, and to a lesser extent community, is vital to coping, it is access to social protection in this context that is fundamental to helping families deal with the burdens caused by maternal mortality. Despite families rallying resources and ensuring care in the short-term, orphaned infants in this high HIV prevalence were at risk of vertically transmitted HIV. Families’ also had difficulty responding to the emotional and psychological needs of children, particularly those who were older at the time of death or as they matured and this had repercussions for the children’s development and longer-term outcomes.

These results illustrate that the extended family provides crucial support and care for children orphaned by deaths from maternal causes. Existing South African research notes that systems of obligation and traditional norms underpin familial support in black South African families [32,33] and the evidence suggests that this research confirms that in a similar way to enabling resilience in households affected by AIDS-related death [34-38] this is also at work in household’s affected by maternal mortality. Historical patterns of adult migration mean that social parenting and caregiving by extended family members are established practices in this context with or without the death of a mother [33,39-41]. Women have a particularly crucial role to play as primary caregivers but face potential consequences as a result. For example, this and other South African research point to the potential for physical implications, for older women especially, but also repercussions for women’s participation in the labour market [42]. This is particularly marked in terms of the impacts of maternal mortality because the most urgent need for care is likely to be for an infant that requires intensive individual and financial commitment and is where adequate care can ensure survival [19,31]. Although the obligation to support and care for family is guided by gender and cultural norms for female care and the placement of children with family based on the mother’s marital status, these results suggest that these norms are adaptable and care is organised for the best interest of children rather than as a result of tradition.

The consequences of maternal mortality are felt broadly within the family, by non-resident members of the child’s extended family (aunts, uncles and paternal grandparents), with implications for the household’s livelihood. The findings indicate that the well-being of the child/ren is the priority of the family and that in most cases traditions are eschewed in favour of the child’s best interest. For example, according to tradition the role of the father and the paternal family is mainly concerned with financial assistance but in this context of high extra-marital fertility paternal families may take on primary care. The importance of fathers’ role in children’s lives is increasingly recognized [43]. Research into the impacts of a general maternal death confirm the findings here in relation to maternal mortality specifically and highlight the need to consider the specific role that father’s play in families affected by maternal death and that despite gender norms, men can be possible caregivers and supportive contributing adults [44-46].

Maternal mortality had complex repercussions at both individual and household levels with the potential to create a financial burden for the wider family. While it is not possible in this analysis to unpack whether the effect of a death from maternal causes specifically would have differential impacts to a maternal death at any other time it is still relevant to note that the impact of maternal mortality would be similar to this affect in the instance of other deaths. The increased, and almost always unexpected, economic and social cost associated with the care of an infant and or children was compounded by the loss of the person who would have been responsible and assisted with this care- the mother.

The system of social protection in South Africa is well developed and it is clear from the families studied here that access to social grants was crucial to their ability to respond to both the needs of the child/ren and to cope with the other expenses associated with a death from maternal causes. While the grant system has a mechanism to directly support orphaned children, it is notable that rather than the foster care grant it was general grants targeted to children and older people in vulnerable families that enabled household coping and were redirected to directly provide for the children at risk. The household-level redistribution and relative importance of social grant income and benefits to those most at risk have been documented in other research in the South African context [32,47-49]. This finding is in contrast to similar research in other contexts where social welfare support were not nearly as robust and where families required greater support [50,51]. Again it is not necessarily possible to differentiate here between the impacts we observed in the instance of a death from maternal causes and maternal death at other times. It does seem fair though to assume the delays and problems, often associated with proving need and eligibility, with accessing a child support or foster care grant for the infant child, documented in this study, are likely to have long-term implications for both families and the surviving infants. The results show that despite systems to support the care of needy infants such as subsidised formula milk, the bulk of respondents did not seem to be aware of these services. Despite the relative importance of access to grants within affected families, they only enabled survival and provided for the most immediate and essential needs of the household and children; the studied families remained relatively poor and vulnerable. It is therefore important that families are adequately informed about and supported to access available support specifically social grants, specifically foster care grants.

In addition to HIV as a potential cause of and risk factor for maternal mortality the results show that children born to HIV-positive mothers who die of maternal causes where transmission is not prevented may be at risk of becoming positive themselves. The findings indicate the need for a close relationship between obstetric and paediatric services to ensure that at risk orphaned infants are screened for HIV after 6 weeks of age and if necessary are directly linked to care and treatment, not only on presentation with symptoms or illness, which appears to be the case in many of the examples from the households enrolled in this study. Quantitative evidence from the Agincourt demographic surveillance site in South Africa suggests that infants whose mothers suffer a death from maternal causes are less likely to survive than those whose mothers live and those whose mothers die from HIV and TB-related causes are 29 time more likely to die [19]. Infants and children qualify for free public provided treatment and care in South Africa and if tested according to policy after six weeks of age, children should therefore be diagnosed and treated and not be diagnosed late or die from HIV-related causes in this context [52,53]. The findings also highlight the need to improve sexual and reproductive health services for HIV-positive women to raise awareness of and reduce the risks involved in pregnancy and childbirth for both the health of the mother and child.

Mothers play an essential role in children’s lives and our findings confirm existing evidence that suggests that South African children without a mother have poorer health, educational [54-56], nutritional [57] and sexual health outcomes, particularly girls [58], compared to children with living mothers. In terms of the stages of the life course the results show the importance of the impact of caring for infants left behind as a result of maternal mortality has implications for the household and caregiver. While families rally to support infants in particular the financial, physical and emotional impacts of a death from maternal causes may result in long-term implications for children orphaned as very small infants. Our findings show that caregivers struggle with providing children with emotional support and care in the period after the death. This is particularly relevant for older children and adolescents in the short-term and as children age into adolescence in the longer term. While assistance from the extended family and access to social grants enables families to cope, their inability to or difficulty with providing sufficient emotional and psychological support to orphaned children was notable. Our findings show that maternal mortality can have ongoing repercussions for children’s development outcomes regardless of life stage.

The study is limited because the generalisation of these qualitative results is limited to residents of this community, although it is possible that certain aspects of the findings may be relevant within other similar contexts in South Africa. It is also important to note that in many ways these results prove to confirm research already conducted within similar communities to explore the implications of maternal death, from HIV at any time. Despite these limitations, these findings provide very valuable and in-depth insights into the cumulative and complex implications of a maternal mortality for infants, children and families within the South African context.

## Conclusion

These results reveal the high costs to surviving infants, children and their families of failing to reduce maternal mortality in South Africa. Despite being relatively well-resourced with very good access to antenatal and skilled birth attendance at health facilities, high levels of maternal mortality persist in South Africa [1]. The response needs to urgently address the preventable causes of maternal mortality by ensuring adequate family planning, antenatal and emergency obstetric care. In particular in a high HIV prevalence context such as South Africa where HIV-positive women are potentially at an increased risk or maternal morbidity and mortality and where prevalence has contributed to slowing progress towards Millennium Development Goal 5 [7,8,17,18,59] joint investment in interventions that address issues of maternal health and HIV are necessary [18]. In addition to highlighting the need to invest to end preventable maternal mortality, [60] these findings indicate that an investment in preventing death from maternal causes is necessary to prevent the economic and social impacts that maternal mortality can have for families. The evidence for these impacts and the way in which households are affected by and respond to maternal mortality suggests that the responses to this issue should not just be about health but also investments in other sectors such as welfare and familial support that enable them to respond.

While families and particularly female family members seem to rally resources in order to provide children with necessary care in this context it is important to ensure that these systems of family support are adequately sustained. Ensuring satisfactory access to and knowledge of social protection is crucial for these children and families. Additional qualitative evidence is needed to explore differential effects for children by gender and to guide future research and inform policies and programs aimed at supporting maternal orphans and other vulnerable children throughout their development.

## Competing interests

The authors have declared that no competing interests exist.

## Author contributions

LK oversaw data collection, performed the primary data analysis and drafted the manuscript. AEY is the study PI, conceived the study and contributed to draft revisions. Both authors read and approved the final manuscript.

## Peer review

Reviewer reports for this article can be found in Additional file 1.

## Supplementary Material

Additional file 1 Click here for file

## Declarations

This article has been published as part of *Reproductive Health* Volume 12 Supplement 1, 2015: True costs of maternal death. The full contents of the supplement are available online at http://www.reproductive-health-journal.com/supplements/12/S1. Publication charges for this supplement were funded by Family Care International and the FXB Center for Health and Human Rights.
