# Supplementary material for: “Without a mother”: caregivers and community members’ views about the impacts of maternal mortality on families in KwaZulu-Natal, South Africa
Source: Reprod Health. 2015 May 6;12(Suppl 1):S5. doi: 10.1186/1742-4755-12-S1-S5 (PMC4423579; doi:10.1186/1742-4755-12-S1-S5)
Supplement: Additional file 1 [file 1742-4755-12-S1-S5-S1.pdf]

Referee's comments to the authors– this sheet <u>WILL</u> be seen by the author(s) and published with the article

Title	"Without a mother": Qualitative findings about the impacts of maternal mortality on families in KwaZulu-Natal, South Africa
Author(s)	Lucia Knight and Alicia Ely Yamin
Referee's name	Sangeetha Madhavan

When assessing the work, please consider the following points, where applicable:

1. Is the question posed by the authors new and well defined?
2. Are the methods appropriate and well described, and are sufficient details provided to replicate the work?
3. Are the data sound and well controlled?
4. Does the manuscript adhere to the relevant standards for reporting and data deposition?
5. Are the discussion and conclusions well balanced and adequately supported by the data?
6. Do the title and abstract accurately convey what has been found?
7. Is the writing acceptable?

Please make your report as constructive and detailed as possible in your comments so that authors have the opportunity to overcome any serious deficiencies that you find and please also divide your comments into the following categories:

- Major Compulsory Revisions (which the author must respond to before a decision on publication can be reached)
- Minor Essential Revisions (such as missing labels on figures, or the wrong use of a term, which the author can be trusted to correct)
- Discretionary Revisions (which are recommendations for improvement but which the author can choose to ignore)

Where possible please supply references to substantiate your comments.

When referring to the manuscript please provide specific page and paragraph citations where appropriate.

General comments: This paper sets out to examine the effects of maternal mortality on well-bring of children and family left behind using qualitative data collect in Kwa-Zulu Natal, South Africa. While the question itself is an important one, I learned very little from this paper. The authors adopt the definition of maternal mortality as defined by WHO – "the death of a woman during pregnancy, childbirth or within 42 days of terminaton." (page 5). However the data and analysis are really about maternal death in general with little to say about maternal mortality. For starters, it would have been useful to have a conceptual framework outline why maternal mortality (as opposed to other forms of maternal death) would be expected to engender unique effects on well-being. Without this, I cannot tell what is different about deaths from maternity related causes vs. deaths of mothers from any cause. They make a vague reference to the link between HIV and maternal mortality on pae 4 with very little elaboration of what these effects are how this might play out in the effect on well-being.

Major compulsory revisions:
Without any conceptual guide, the data presented in the forms of quotes are not particularly informative and provide no new insights about the role of extended kin and the effects on children. Instead, it is essential a long list of quotes with very little critical analysis of any of them. As common with the presentation of qualitative data, the auhors favor quantity over quality which usually does little to improve our

(continue on the next sheet)

Continued:
understanding about these processes.

In sum, unless the authors can show me why they think maternal mortality is different in its effect from other causes of death, I find little that is new in this paper.

Referee's comments to the authors– this sheet WILL be seen by the author(s) and published with the article

Title	"Without a mother": Qualitative findings about the impacts of maternal mortality on families in KwaZulu-Natal, South Africa
Author(s)	Lucia Knight and Alicia Ely Yamin
Referee's name	Wanga Zembe

When assessing the work, please consider the following points, where applicable:

1. Is the question posed by the authors new and well defined?
2. Are the methods appropriate and well described, and are sufficient details provided to replicate the work?
3. Are the data sound and well controlled?
4. Does the manuscript adhere to the relevant standards for reporting and data deposition?
5. Are the discussion and conclusions well balanced and adequately supported by the data?
6. Do the title and abstract accurately convey what has been found?
7. Is the writing acceptable?

Please make your report as constructive and detailed as possible in your comments so that authors have the opportunity to overcome any serious deficiencies that you find and please also divide your comments into the following categories:

- Major Compulsory Revisions (which the author must respond to before a decision on publication can be reached)
- Minor Essential Revisions (such as missing labels on figures, or the wrong use of a term, which the author can be trusted to correct)
- Discretionary Revisions (which are recommendations for improvement but which the author can choose to ignore)

Where possible please supply references to substantiate your comments.

When referring to the manuscript please provide specific page and paragraph citations where appropriate.

General comments:
Interesting paper with stirring excerpts from participants, addressing an under-researched yet important topic in South Africa and similar settings with high maternal mortality rates and high HIV prevalence.

Major compulsory revisions: none

Minor essential revisions:
1. Authors need to consider revising the title of the paper to emphasize the fact that they conducted the study with caregivers and community members, as the paper does not at all include the views of orphans. The current title leads the reader to expect to hear the perspectives of orphans as well. A revised title could be something along the lines of ""Without a mother": caregivers and community members' views about the impacts of maternal mortality on families in KwaZulu-Natal, South Africa"
2. Lines 79-80 –reference? I know that poverty estimates at small area level in SA are scarce, but if this ref exists, please insert it.

3. Line 91 "The families captured in the study generally sit on or below the poverty line" How was this established?

Continued: 4. Line 103 –were the interviews back-translated?
5. Line 104 –what framework are the authors referring to?
6. Line 177 –multiple orphan siblings
7. Line 194 – begin sentence with "in such cases, even...."
8. Line 228 – "proof of deaths" should be singular
9. Line 245 –specify that it is formula milk
10. Line 277 –remove double full stop
11. Line 288 – "ask what happen" –happened
12. Line 292-293 "They do attend school though their performance is not the same and that sometimes prompts you to follow up on the problem of child" replace with "the child"
13. Line 310-311 "want her to learn and finish [grade] 10 but now since she has a boyfriend…*"*. I am pretty sure this is not a grade, but rather 'standard' 10 (matric).

14. In the Discussion the authors make some strong concluding statements, which I agree with, but which I feel would benefit from more data to support the findings. For instance in Line 320 the authors find that "it is access to social protection in this context that is fundamental to helping families deal with the burdens caused by maternal death", I think the paper would benefit greatly from additional quotes to support such a big and important finding. Presently there are only 2 quotes related to role played by grants, (the other 2 are on barriers to accessing the foster care grant).

15. Line 342-343 –consider revising sentence, suggestion: "For example, according to tradition the role of the father and the paternal family is mainly concerned with financial assistance but in this context of high extra-marital fertility paternal families may take on primary care"

16. Line 362 - Would you not also add that the foster care grant process needs to be considerably simplified and that all orphaned children, including those who have lost one parent, should be able to access this important grant?

17. Line 368-370 -Presumably young children as opposed to older children? What were the age groups in the Agincourt study?

18. Line 371-372 -This sentence "Infants and children qualify for free public provided treatment and care in South Africa" does not fit well here, consider taking it elsewhere, unless the point the authors are trying to make is that even though infants and children qualify for free treatment and care, those whose mothers die (especially from HIV and TB related causes) are at greater risk of dying?

19. Line 398-401 – The two sentences address two separate issues. As I see it the first sentence addresses the need for maternal death to be prevented, and the second sentence is addressing the need for the impact of maternal death to be ameliorated? In other words the second sentence is saying where maternal deaths do occur investments in other sectors such as welfare and familial support are needed to ensure that the impact on those left behind is minimized?

20. Line 403 - repeated use of the word 'necessary' in the same sentence

Discretionary revisions: none

Supplement Editors' comments

As you can see one of the reviewers raised serious concerns, but we estimate that you manuscript merits to be consider for publication. We wonder if you can provide an answer to these concerns.
One reviewer mentions about the need to edit the manuscript carefully for correct grammar and sentence construction. Please see if you can address this recommendation.
We consider that the second sentence of the abstract involved conclusions that are beyond the results provided in your study. We wonder if you can delete this sentence and instead to focus on conclusions derived from your study
